# Valorization of Water Lettuce (*Pistia stratiotes* L.) Through Bioconversion for Black Soldier Fly Larvae (*Hermetia illucens*): Larvae Growth, Survival Rate, and Nutritional Quality

**DOI:** 10.3390/insects16101068

**Published:** 2025-10-20

**Authors:** Juste Vital Vodounnou, Romaric Iko, Rendani Luthada-Raswiswi, Sèlomè Wilfried Sintondji, Cayen Sédro Alofa, Gildas Djidohokpin, Farokh Niass, Jean-Claude Micha

**Affiliations:** 1 Research Unit in Aquaculture and Fisheries Management (URAGEP), Ecole d’Aquaculture, Université Nationale d’Agriculture (UNA), Kétou BP 43, Benin; romariciko@gmail.com; 2 Key Laboratory of Freshwater Aquatic Genetic Resources, Ministry of Agriculture and Rural Affairs, Shanghai Ocean University, Shanghai 201306, China; 3 School of Life Sciences, College of Agriculture, Engineering and Science, Pietermaritzburg, University of KwaZulu Natal, Scottsville 3209, South Africa; luthada-raswiswir@ukzn.ac.za; 4 Laboratory of Hydrobiology and Research on Wetlands, (LHyReZ) of Abomey-Calavi University (UAC), Cotonou BP 526, Benin; sintondjiwilfried@gmail.com (S.W.S.); cayen.alofa@uac.bj (C.S.A.); gdjidohokpin@gmail.com (G.D.); 5 Department of Natural Resources Management, Faculty of Environmental Sciences (FSE), University of N’zérékoré (UZ), N’Zérékoré BP 50, Guinea; 6 Complex Systems Modeling and Biological & Agronomic Sciences Laboratory (LABAM), Gaston Berger University, Saint-Louis BP 234, Senegal; farokh.niass@ugb.edu.sn; 7 Research Unit in Environmental and Evolutionary Biology (URBE), Institute of Life, Earth and Environment (ILEE), University of Namur, Rue de Bruxelles 61, 5000 Namur, Belgium; jean-claude.micha@unamur.be

**Keywords:** water lettuce, black soldier fly larvae, bioconversion, substrate

## Abstract

The rapid proliferation of water lettuce, *Pistia stratiotes*, poses significant ecological challenges. This study investigates the use of *Pistia stratiotes* as a substrate for producing black soldier fly larvae. The experiment used 40 g of black soldier fly larvae (BSFL) pupae for all five substrates (T0, T25, T50, T75, and T100). Each treatment had three replicates. The physicochemical parameters, such as the temperature and pH, ranged from 27.30 °C to 27.40 °C and from 7.03 to 7.60, respectively. The survival rates ranged from 90.13% to 94.73%. The biochemical parameters showed significant differences, including dry matter, organic matter, ash, crude protein, crude lipid, crude fiber, carbohydrate, and energy (*p* < 0.05). Regarding the larvae’s growth and substrate use, the BSFL production varied depending on the rearing substrate. The highest growth was observed in substrates without water lettuce leaf (WLL) (T0), followed by those in treatment at 25% (T25) and 50% (T50) inclusion of WLL and then the substrate made solely of WLL (T100). The substrates degradation rate in this study varied significantly, ranging from 46.40% to 67.38%. The physicochemical characteristics of the substrates are associated with the growth performance of the BSFL, and the inclusion of WLL influenced the nutritional composition of the larvae.

## Simple Summary

The invasive aquatic plant *Pistia stratiotes*, commonly known as water lettuce, is recognized as a significant threat to ecosystems. Its presence can adversely affect biodiversity and disrupt aquatic habitats. This study aimed to explore an innovative solution: converting this plant into a sustainable resource through the cultivation of black soldier fly larvae, which can be used as clean feed for aquaculture. The objectives were to determine the growth, survival rates, and nutritional quality of the black soldier fly larvae fed with different levels of water lettuce leaf (WLL). The results showed that a higher level of WLL had a significant effect on the growth and nutritional quality of the black soldier fly larvae. However, the survival rates were not significantly affected by different ratios of the WLL. We concluded that WLL can be used as a substrate for cultivating black soldier fly larvae, offering a valuable option for fish farmers seeking an alternative protein source to enhance production and profits while reducing fish feed costs.

## 1. Introduction

Black soldier flies (*Hermetia illucens* (L. 1758)) currently represent a powerful tool for the decomposition and valorization of organic waste [1,2]. They enable the recycling of organic waste by transforming it into food for their larvae [3,4]. Considering projections, whereby the world population is expected to reach 9 billion people in 2050 [5], promoting responsible production practices based on the recycling and valorization of organic waste becomes essential. Due to its powerful bioconversion capacity, the black soldier fly has attracted the attention of several researchers, not only for its environmental benefits but also for its valorization in animal production and aquaculture. The larvae of black soldier flies serve as a valuable source of protein for animal production and aquaculture [6,7]. The protein content of *H. illucens* varies from 37% to 63% of dry matter, while the lipid content ranges from 7% to 39%, depending on the rearing substrates [8,9]. They have a well-balanced profile of essential amino acids (EAA), comparable with fish meal [8,10,11]. A wide variety of organic wastes from animal and plant have been used as substrates for BSFL production, including kitchen scraps, animal manure, oilseed cakes, bran, and municipal waste [12,13,14]. Recently, in the effort to combat the proliferation of invasive aquatic plants, black soldier fly larvae have been used to valorize water hyacinth leaves [15]. Invasive aquatic plants pose significant challenges to aquatic ecosystems, as obstacles to navigation and fishing, and substantially reduce aquatic biodiversity [16,17,18]. They also increase the evapotranspiration of water bodies and contribute to the formation of microhabitats that host several disease vectors [17,19]. Water lettuce, also known as *Pistia stratiotes*, is one such invasive plant, commonly found in freshwater ecosystems. It is a fast-growing species that can double its biomass every 15 days, depending on the temperature and nutrient availability [20,21]. It is a stemless plant that forms a rosette 5 to 25 cm wide, with an extensive network of fibrous roots submerged in water. Native to South America, it has spread across the globe, especially in Africa [20,22]. Several studies have focused on its eradication through biological and chemical control methods without achieving long-term success. Chemical control can lead to environmental issues due to the presence of chemical residues. Other uses should be explored to effectively restrict the biomass of *Pistia stratiotes* in waterways. It can be used as a fertilizer to enhance soil fertility. Water lettuce has also been explored as a potential feed for livestock; however, its high fiber and low protein content require supplementation and processing. It is in this context that the valorization of *Pistia stratiotes* as a substrate to produce black soldier fly larvae has been considered due to the larvae’s significant capacity to be used as an alternative protein source to replace the most expensive ingredient, fish meal, in aquaculture feeds and reduce the cost of feed while increasing the production.

## 2. Materials and Methods

### 2.1. Experimental Location

The work was carried out on a farm in the Plateau department of the Sakété district in Benin. The farm, ANIMAUX DIVIN, specializes in producing black soldier fly larvae.

### 2.2. Obtaining Black Soldier Fly Larvae

Forty grams of black soldier fly pupae were placed in optimal conditions to produce black soldier fly larvae (BSFL), as described by [23]. After the pupae metamorphosed into adults, they began to reproduce by laying eggs in a breeding enclosure. Rotten mango fruits served as an attractant, and the eggs were deposited on specially designed devices based on a model [23]. The harvested neonate larvae were incubated in a chick feed substrate for five days to promote their normal development before being utilized in the experiments [9,24].

### 2.3. Rearing Substrates

Five production substrates (T0, T25, T50, T75, and T100) were utilized in this study. The control feed (T0), currently used on the farm to produce black soldier fly larvae, consists of an equal mixture of brewer’s spent grain, wheat bran, palm kernel cake, and soy bran obtained from soy cheese processing. The other substrates are T25 (75% T0 and 25% of WLL), T50 (50% T0 and 50% of WLL), T75 (25% T0 and 75% of WLL), and T100 (100% of WLL). *Pistia stratiotes* were harvested from a pond near a tributary of the Ouémé River. To gain a comprehensive understanding of the nutritional characteristics of water lettuce, the roots and the leaves were carefully analyzed using the methods outlined by the Association of Official Analytical Chemists [25]. These parts were carefully separated and dried before analysis. Key parameters, organic matter, ash, crude protein, crude lipid, and crude fiber were then measured and recorded, as detailed in Table 1. The leaves were incorporated into the experimental diets due to their relatively high protein content. Before inclusion, they were sun-dried for seven days, ground, and sieved through a 1 mm mesh. The source water, from which the water lettuce leaf was derived, has not been reported to contain heavy metals or other hazardous contaminants. Consequently, it assumed that the WLL were free from any heavy metal contamination. The leaves were sun-dried for seven days, ground, and sieved through a 1 mm mesh. The composition of the experimental substrates is presented in Table 2. We measured the dry matter, organic matter, ash, total nitrogen, crude protein, crude lipid, crude fiber, and carbohydrate content. Subsequently, the energy content of each substrate was calculated in kcal/kg (Table 2).

### 2.4. Experimental Design and Follow-Up

To obtain a homogeneous sample, the larvae collected after incubation were sorted using two sieves with mesh sizes of 2 mm and 1 mm [24]. The larvae retained in the 1 mm sieve, after an initial inclusion of larvae retained in the 2 mm sieve, were used in the experiment. Five experimental substrates (T0, T25, T50, T75, and T100) were tested in the study. The experimental setup included fifteen tanks (measuring 25 cm × 14 cm × 12 cm), arranged in triplicate for each treatment. The seeding density was set at 1 larva per gram of substrate [14,23]. There were 1500 larvae per tank. The substrates were moistened to a level of 70% humidity [9]. The observations were conducted over 10 days, with growth monitored at two-day intervals. A 10% sample from each substrate was weighed using a precision scale with a sensitivity of 0.001 g. Samples from each tank were weighed collectively and subsequently returned to their respective substrates following biomass collection. During the experimental period, physicochemical parameters, including the pH and temperature of the substrates, were measured using a pH meter and a thermometer, with measurements taken twice daily at 7:00 a.m. and 5:00 p.m.

### 2.5. Larvae Harvest

At the end of the experiment, all the larvae were collected from their original tanks. They were then dried in an oven at 105 °C for six hours according to AOAC methods [25]. Depending on the treatment assigned to each group, the harvested larvae were dried to an equilibrium water content and then analyzed in the laboratory for their protein, lipid, and dry matter content. The remaining substrates were also dried in an oven at 105 °C for 6 h, as per the specific treatments, and then weighed to evaluate the substrate loss.

### 2.6. Chemical Analysis of the Larvae Samples

The pH value was measured by inserting pH paper (universal pH paper) into the substrate. The dry matter, organic matter, ash, and crude protein of the black soldier flies were determined using the methods described by [25,26].

### 2.7. Growth Performance and Survival Rate of the BSLF and the Substrate Utilization Estimates

The following growth performance estimates were calculated using the formulas described by [27] to assess the performance of the substrates: survival rate (SR), daily weight gain (DWG), production (P), and degradation rate (DR).

SR (%) = 100 × FN/IN,

where IN represents the initial number of larvae, and FN represents the final number of larvae.

DWG (g/d) = (FBW − IBW)/∆T,

where IBW represents the initial biomass weight (g), FBW represents the final biomass weight (g), and Δt represents the duration of the experiment in number of days.

P (g of larvae/kg of substrate) = (FB − IB)/Q,

where Q is the quantity of substrate (kg).

DR (%) = [(W − R)/W] × 100,

where W is the initial weight of the substrate, and R is the residual weight of the substrate.

### 2.8. Data Analysis

The data were organized using Excel software. Various parameters, including zootechnical, physicochemical, substrate utilization, and hematological, were calculated using the formulas described in Section 2.7. The mean and standard deviation were calculated for each variable. The data were analyzed using a one-way analysis of variance (ANOVA) in STATVIEW version 5.01 software, following verification of the variance homogeneity with Hartley’s test. Before that, the Shapiro–Wilk test was conducted to check normality.

Significant differences between the means were assessed using Fisher’s test, with a significance level set at *p* = 0.05.

## 3. Results

### 3.1. Growth, Survival Rate, and Substrate Utilization Estimates

**Figure 1:**
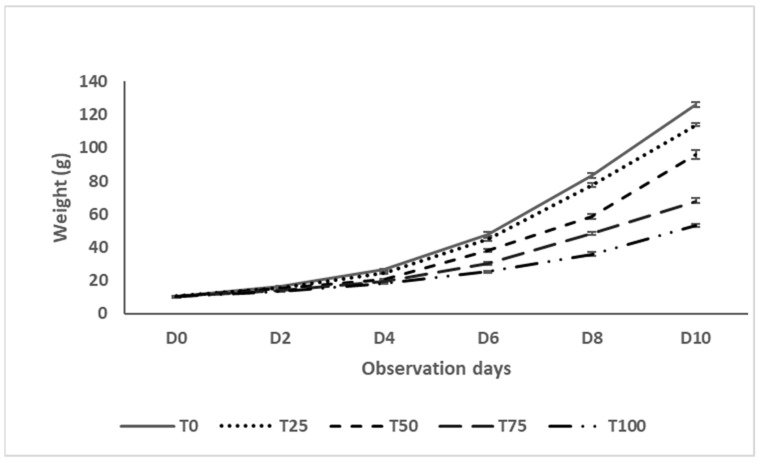
Growth curve of BSFL in various substrates of production based on water lettuce.

**Figure 2:**
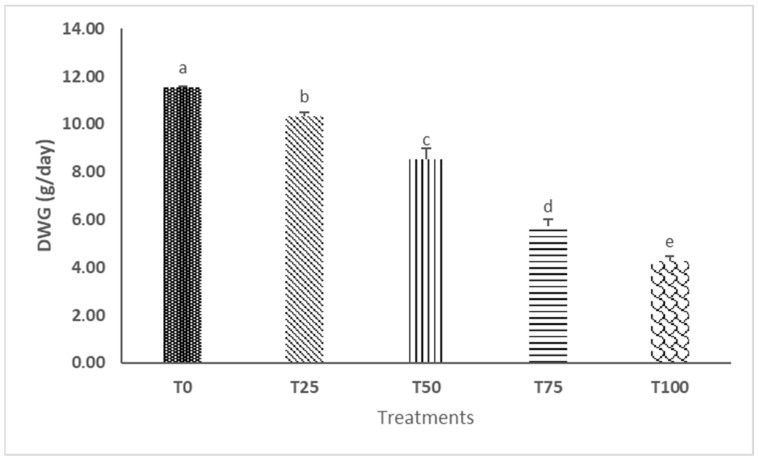
BSFL daily weight gain in the various substrates of production.

The growth curves (Figure 1) of black soldier fly larvae (BSFL) production varied based on the rearing substrates used. Adding more water lettuce leaf (WLL) resulted in a lower weight for the black soldier fly larvae. This trend was also evident in the daily weight gain (DWG). A significant difference was observed in the DWG between the different treatments (*p* < 0.05). Larvae reared on the substrate without WLL (T0) exhibited the highest weight gain, followed by T25, T50, and T75, while those in the T100 showed the lowest DWG. Notably, the DWG declined significantly when the WLL incorporation exceeded 50% (Figure 2).

Similarly to the patterns observed in the daily weight gain, both the final biomass and production of black soldier fly larvae (BSFL) differed significantly between the treatments. The highest final biomass was in the substrate without WLL, T0, followed by T25, T50, and T75, with the lowest at T100 (Table 3, *p* < 0.05). A comparable trend was noted for BSFL production as well as for the substrate degradation rate (Table 3). In contrast, the survival rate of BSFL did not differ significantly between treatments (*p* > 0.05), ranging from 90.13% in T100 to 94.73% in T75.

### 3.2. Nutritional Quality of black Soldier Fly Larvae (BSFL) from Different Substrates

Significant differences were observed between the rearing substrates (*p* < 0.05) regarding their impact on the nutritional quality of BSFL. The use of WLL in the production of BSFL affected the nutritional composition of the larvae. Significant differences (*p* < 0.05) were found between the treatments in organic matter, ash, fiber, lipid, and protein content (Table 4). The organic matter content ranged from 87.33% in T100 to 90.16% in T0, while the ash content varied from 9.83% in T0 to 12.66% in T100. The lipid content ranged from 15.00% in T100 to 31.40% in T0, and the fiber content ranged from 17.11% in T100 to 20.41% in T0. No significant differences (*p* > 0.05) were observed in the dry matter content of the BSFL between treatments.

## 4. Discussion

Black soldier fly larvae are polyphagous detritivores known for their high feed conversion rate, which makes them suitable for converting various animal and plant organic materials into valuable insect-derived proteins and lipids [28,29]. These larvae are increasingly being studied as an innovative and sustainable ingredient for aquaculture and animal feed [30,31]. The current study results demonstrate that water lettuce leaf can be used in conjunction with other substrates and is suitable for producing black soldier fly larvae.

### 4.1. Growth, Survival Rate, and Substrate Utilization Estimates

The survival rate of black soldier fly larvae (BSFL) showed no significant differences (*p* > 0.05), indicating that the inclusion of water lettuce leaf (WLL) did not adversely affect larval survival, even though the performance was lower in the treatment consisting solely of WLL. These results suggest that WLL is non-toxic to BSFL production. BSFL are known to be highly sensitive to environmental toxicity and cannot survive or thrive in unfavorable conditions [32,33,34]. This finding contrasts with that of [15], who reported significant effects on survival when different ratios of water hyacinth (*Eichhornia crassipes*) leaves were used as substrate. The growth of BSFL and the degradation rate of the substrate are interconnected. It has been observed that substrates with higher protein and organic matter content support both larval growth and substrate decomposition.

The energy required for BSFL survival and reproduction is derived from the nutritional reserves accumulated during their larval development [35,36,37]. The significant differences (*p* < 0.05) in growth and substrate utilization were attributed to the quality and composition of the production substrates. Previous studies also highlight the importance of the substrate nutritional composition for BSFL growth, nutritional quality, and adult reproduction [37,38]. Significant differences were observed between treatments for all growth and substrate utilization estimates (FB, DWG, P, and DR). These results are similar to those reported by [15], who used water hyacinth (*Eichhornia crassipes*) leaves as a substrate. However, the differences observed were not due to the temperature and pH of the substrate. The substrate’s temperature (27.30 °C (T0) to 27.40 °C (T100)) and pH 7.03 (T25) to 7.60 (T100) ranges were within the recommended range (24-36 °C and 6-8, respectively) for optimal development of the BSFL [14]. Differences in FB, DWG, P, and DR were apparently due to the variation in substrate composition. When comparing the present study to a study using water hyacinth leaves at 100% substitution, the BSFL degraded water lettuce leaves more efficiently (46.40%) than water hyacinth leaves (42.93%) [15]. Substrates rich in organic matter, nitrogen, and lipids enhance reasonable energy reserves and growth in black soldier fly larvae [39,40,41]. Meanwhile, substrates rich in carbohydrates prolong the development time of the BSFL [42]. Furthermore, protein-to-carbohydrate ratios of 1:2 to 1:3 result in the highest larval yield, utilizing food resources for BSFL production while meeting the larvae’s macronutrient needs. Our study supports the findings reported by [42], which indicate that protein-to-carbohydrate ratios of 1:4 and 1:5 in treatments T75 and T100 did not yield good results compared with the treatments with ratios within the recommended range.

### 4.2. Nutritional Quality of Black Soldier Fly Larvae (BSFL) Produced Using Substrates Made from Water Lettuce Leaves

Significant differences (*p* < 0.05) were observed between the treatments regarding organic matter content, fibers, lipids, and proteins. As the incorporation rate of water lettuce increased, the nutritional quality of the BSFL decreased. However, the protein content of the BSFL produced in this study generally remained within the range (37 to 63%) described by [8,9], except for treatment T100, which was made solely of water lettuce leaves, where the protein content was 33.00%. The protein contents in treatments T0 (42.96%) and T25 (42.16%) were slightly higher than those reported by [23], which was around 40%, and similar to those studies by [14] that involved 25% water hyacinth leaves’ incorporation. In contrast, the BSFL produced with 100% water hyacinth leaves exhibited a slightly lower protein content of approximately 31%, compared with those made solely with water lettuce leaf containing 33% protein. The lipid content followed a similar pattern, with BSFL lipid levels remaining within the range (7–39%) described by [8,9,40,41]. This study confirms that the type of substrate significantly affects the nutritional quality of BSFL, as previously concluded by various researchers [42,43]. It is recommended that water lettuce be harvested from clean water to prevent potential toxin contamination, as the plant can accumulate heavy metals [44].

## 5. Conclusions

The present study evaluated the effects of water lettuce leaf on the growth performance, survival, and substrate decomposition of the BSFL, as well as on the nutritional quality of the larvae. It showcases the environmental management of invasive plants and promotes sustainable protein production for aquaculture. The water lettuce leaf can be used to produce BSFL without negatively affecting their survival. Inclusion levels above 50% significantly reduce the performance, while low inclusions (≤25%) may be sustainable. This study highlights that the nutritional quality of BSFL is greatly affected by the quality of the production substrates. Sustainable use of water lettuce leaf in BSFL production depends on the availability and proper harvesting of *Pistia stratiotes* from clean water sources.

## Figures and Tables

**Table 1:** Nutritional composition of water lettuce collected from a pond near the Ouémé River.

Parameters	Plant Part	
	Leaves	Roots
Organic matter (%)	63.25 ± 0.22	54.85 ± 0.19
Ash (%)	36.74 ± 0.14	45.15 ± 0.16
Crude protein (%)	7.12 ± 0.10	3.08 ± 0.12
Crude lipids (%)	2.8 ± 0.08	1.74 ± 0.05
Crude fiber (%)	18.11 ± 0.11	21.6 ± 0.09

Values are expressed as mean ± standard deviation.

**Table 2:** Formulation and physico-chemical and biochemical composition of the substrates used for black soldier fly larvae production.

Ingredients	T0	T25	T50	T75	T100	F-Value	*p*-Value
Farm Feed for BSFL	100	75	50	25	0	-	-
Water Lettuce LeaF	0	25	50	75	100	-	-
**Total**	100	100	100	100	100	-	-
Parameters for the Physio-chemical and Biochemical Compositions of the Substrates							
pH	7.56 ± 0.12 a	7.03 ± 0.39 a	7.06 ± 0.21 a	7.13 ± 0.43 a	7.60 ± 0.15 a	0.91	0.49
Dry Matter (%)	92.30 ± 0.12 a	91.70 ± 0.36 a	91.46 ± 0.17 ab	90.70 ± 0.40 b	89.66 ± 0.20 c	13.37	0.0005
Organic Matter (%)	70.46 ± 0.17 a	68.87 ± 0.01 b	66.95 ± 0.01 c	65.02 ± 0.01 d	63.19 ± 0.04 e	1284.14	<0.0001
Ash (%)	29.43 ± 0.17 a	31.05 ± 0.01 b	33.00 ± 0.01 c	34.96 ± 0.01 d	36.80 ± 0.04 e	1321.52	<0.0001
Crude Protein (%)	21.30 ± 0.11 a	17.77 ± 0.01 b	14.22 ± 0.01 c	10.70 ± 0.01 d	7.15 ± 0.02 e	11,179.52	<0.0001
Crude Lipids (%)	7.18 ± 0.16 a	5.89 ± 0.01 b	4.76 ± 0.01 c	3.70 ± 0.01 d	2.76 ± 0.08 e	451.89	<0.0001
Carbohydrates (%)	44.63 ± 0.31 a	43.78 ± 0.01 b	42.34 ± 0.01 c	40.94 ± 0.01 d	39.53 ± 0.37 e	88.78	<0.0001
Crude Fiber (%)	12.81 ± 0.03 a	14.20 ± 0.01 b	15.51 ± 0.01 c	16.83 ± 0.01 d	18.15 ± 0.03 e	8185.28	<0.0001
Protein/Carbohydrate Ratio	0.47 ± 0.18 a (≃1:2)	0.40 ± 0.01 b (≃1:2.5)	0.33 ± 0.04 c (≃1:3)	0.26 ± 0.03 d (≃1:4)	0.18 ± 0.05 e (≃1:5)	869.66	<0.0001
Energy (kcal/kg)	3539.90 ± 12.61 a	3276.36 ± 1.10 b	3001.73 ± 1.51 c	2735.46 ± 1.12 d	2479.39 ± 20.94 e	1468.01	<0.0001

Values are expressed as mean ± standard deviation. Values in the same line with a letter in common are not significantly different (*p* > 0.05). T0 (100% Farm Feed for BSFL (used as a control), T25 (75% T0 and 25% WLL), T50 (50% T0 and 50% WLL), T75 (25% T0 and 75% WLL), and T100 (100% WLL).

**Table 3:** Growth performance and substrate utilization of *P. stratiotes* valorization in black soldier fly larvae (BSFL) production.

Parameters	T0	T25	T50	T75	T100	F-Value	*p*-Value
Initial biomass weight (g)	10.55 ± 0.01 ^a^	10.56 ± 0.01 ^a^	10.56 ± 0.01 ^a^	10.57 ± 0.01 ^a^	10.57 ± 0.01 ^a^	0.61	0.66
Final biomass weight (g)	126.00 ± 0.26 ^a^	113.83 ± 0.88 ^b^	95.73 ± 2.66 ^c^	67.83 ± 1.72 ^d^	53.24 ± 1.08 ^e^	381.35	<0.0001
Survival rate (%)	92.66 ± 1.76 ^a^	93.00 ± 1.52 ^a^	93.33 ± 1.33 ^a^	94.73 ± 0.26 ^a^	90.13 ± 2.82 ^a^	1.061	0.424
Production (g/kg substrate)	92.35 ± 0.22 ^a^	82.62 ± 0.71 ^b^	68.13 ± 2.14 ^c^	45.80 ± 1.38 ^d^	34.14 ± 0.87 ^e^	380.39	<0.0001
Degradation rate (%)	67.38 ± 0.69 ^a^	62.18 ± 1.17 ^b^	60.16 ± 0.79 ^b^	53.20 ± 1.00 ^c^	46.40 ± 1.22 ^d^	67.21	<0.0001

Values are expressed as mean ± standard deviation. The values in the same line with a letter in common are not significantly different (*p* > 0.05). T0 (100% Farm Feed for BSFL (used as a control)), T25 (75% T0 and 25% WLL), T50 (50% T0 and 50% WLL), T75 (25% T0 and 75% WLL)*,* and T100 (100% WLL).

**Table 4:** Nutritional values of black soldier fly larvae produced using different substrates.

Parameters	T0	T25	T50	T75	T100	F-Value	*p*-Value
Dry Matter (%)	39.75 ± 0.80 ^a^	39.67 ± 0.64 ^a^	39.83 ± 0.33 ^a^	40.44 ± 0.39 ^a^	39.57 ± 0.82 ^a^	0.28	0.87
Organic Matter (%)	90.16 ± 0.44 ^a^	89.50 ± 0.88 ^a^	89.00 ± 0.57 ^a^	88.00 ± 0.57 ^b^	87.33 ± 0.88 ^b^	3.76	0.04
Ash (%)	9.83 ± 0.44 ^a^	10.50 ± 0.29 ^a^	11.00 ± 0.57 ^ab^	12.00 ± 0.57 ^b^	12.66 ± 0.88 ^b^	3.76	0.04
Crude Protein (%)	42.96 ± 0.31 ^a^	42.16 ± 0.72 ^ab^	40.16 ± 0.44 ^b^	38.16 ± 0.44 ^c^	33.00 ± 1.15 ^d^	33.70	<0.0001
Crude Lipids (%)	31.40 ± 0.30 ^a^	31.00 ± 0.57 ^a^	27.00 ± 2.08 ^b^	21.00 ± 1.00 ^c^	15.00 ± 1.15 ^d^	38.30	<0.0001
Crude Fiber (%)	20.41 ± 0.30 ^a^	19.86 ± 0.46 ^a^	19.16 ± 0.93 ^a^	18.33 ± 0.60 ^b^	17.11 ± 0.19 ^b^	5.40	0.01

Values are expressed as mean ± standard deviation. Values in the same line with a letter in common are not significantly different (*p* > 0.05). T0 (100% of Farm Feed for BSFL (used as a control), T25 (75% T0 and 25% WLL), T50 (50% T0 and 50% WLL), T75 (25% T0 and 75% WLL), and T100 100% WLL).

## Data Availability

The data used and/or analyzed during the current study are available from the corresponding author upon reasonable request.
